# Optimizing the maximum reported cluster size in the spatial scan statistic for ordinal data

**DOI:** 10.1371/journal.pone.0182234

**Published:** 2017-07-28

**Authors:** Sehwi Kim, Inkyung Jung

**Affiliations:** Department of Biostatistics and Medical Informatics, Yonsei University College of Medicine, Seoul, Korea; Stony Brook University, Graduate Program in Public Health, UNITED STATES

## Abstract

The spatial scan statistic is an important tool for spatial cluster detection. There have been numerous studies on scanning window shapes. However, little research has been done on the maximum scanning window size or maximum reported cluster size. Recently, Han et al. proposed to use the Gini coefficient to optimize the maximum reported cluster size. However, the method has been developed and evaluated only for the Poisson model. We adopt the Gini coefficient to be applicable to the spatial scan statistic for ordinal data to determine the optimal maximum reported cluster size. Through a simulation study and application to a real data example, we evaluate the performance of the proposed approach. With some sophisticated modification, the Gini coefficient can be effectively employed for the ordinal model. The Gini coefficient most often picked the optimal maximum reported cluster sizes that were the same as or smaller than the true cluster sizes with very high accuracy. It seems that we can obtain a more refined collection of clusters by using the Gini coefficient. The Gini coefficient developed specifically for the ordinal model can be useful for optimizing the maximum reported cluster size for ordinal data and helpful for properly and informatively discovering cluster patterns.

## Introduction

Spatial cluster detection is an important tool in spatial epidemiology to identify areas having unusually high or low rates of disease outcome. The spatial scan statistic is one of the commonly used methods based on various models such as Bernoulli, Poisson [1], ordinal [2], exponential [3], multinomial [4], and normal [5, 6]. The method has been implemented with the freely available software SaTScan^™^ (www.satscan.org), which makes the spatial scan statistic more accessible [7]. When using the software to detect spatial clusters, users can define the scanning window shapes, circle or ellipse, and the maximum scanning window size. Numerous studies have compared the performance of different window shapes [8–10]. On the other hand, the issue on determining the maximum scanning window size (MSWS) or the maximum reported cluster size (MRCS) has received relatively less attention.

In many studies, the scanning window size is set to a maximum of 50% of the total population. The spatial scan statistic evaluates all possible windows up to the maximum and finds the most likely cluster with the highest value of the test statistic. In addition to the most likely cluster, there can be secondary clusters with high values of the test statistic. Reporting all of them may not be informative because many of them would overlap with the most likely cluster or each other. Also, multiple smaller clusters which may be more meaningful can be concealed by a single larger cluster because the single cluster consisting of the multiple clusters and insignificant neighboring areas may have a higher value of the test statistic. In SaTScan software, there are advanced output features of criteria for reporting secondary clusters and of setting the maximum reported spatial cluster size. The default setting for these two options is reporting non-overlapping secondary clusters and setting the MRCS at 50%. However, there is no rationale for this, and the reported clusters could be larger than the true clusters including less informative areas [11]. Riberio and Coasta [12] conducted an extensive simulation study to evaluate the effect of the maximum scanning window size and found that performance can be sensitive to the maximum window size. As Han et al. [13] pointed out, however, users should never run the analysis multiple times using different values for the MSWS. The results of such analyses would suffer from the multiple testing problem. Instead of trying to find an optimal MSWS, we can rerun the analysis and request that the SaTScan software only report clusters of a certain maximum size, while still adjusting for the multiple testing inherent in all the sizes considered in the other prior analyses of the same data, by keeping the MSWS fixed at a larger value [13]. Still, in earlier versions of SaTScan, there was no criterion for choosing the best MRCS.

Recently, to determine optimal cluster reporting sizes, Han et al. [13] proposed using the Gini coefficient [14] as a measure to assess the degree of heterogeneity of a cluster model. Through a simulation study, they found that the Gini coefficient can identify a more refined collection of non-overlapping clusters to report. They also showed that the Gini coefficient satisfies important theoretical features such as being invariant under a uniform multiplication of the population numbers by the same constant. The method has been implemented in the SaTScan^™^ software version 9.3 or later. However, the criterion was evaluated for the Poisson model only, and the applicability to other models has not been tested yet.

In this paper, we adopt the Gini coefficient to be applicable to the ordinal model proposed by Jung et al. [2]. The ordinal model can be used for spatial cluster detection of ordinal data such as cancer stage or grade. It has been applied to various fields, for example, Bell et al. [15] explored the spatial distribution of quality of life outcomes after pediatric injury; Fuchs et al. [16] searched for spatial clusters in mountain hazards using a damage ratio outcome classified as high, medium, and low; and Westercamp et al. [17] examined spatial clusters with or low rates of higher education levels. In the following section, we briefly review the spatial scan statistic for count and ordinal data and provide descriptions of the Gini coefficient for optimizing MRCS in the Poisson-based spatial scan statistic for count data. From there, the application of the optimization criterion for the ordinal model is proposed. We evaluate the performance of the criterion via a simulation study and provide a real data example. We discuss our findings and present concluding remarks in the final section.

## Methods

### Spatial scan statistic

The spatial scan statistic is based on the likelihood ratio test, and statistical significance is evaluated using Monte Carlo hypothesis testing [18]. This method makes it possible to detect clusters where the distribution of events (e.g. disease prevalence, incidence, and mortality) differs from that of the surrounding areas. For this, a large number of candidate areas (scanning windows) are formed in a pre-defined window shape with various sizes up to a maximum size. Then, the likelihood ratio test statistic is used to compare each of the candidate areas with the outside area. The area with the maximum test statistic value defines the most likely cluster, and the areas that are able to reject the null hypothesis on their own strength define the secondary clusters. In SaTScan^™^, 50% of the total population is the default option for the maximum scanning window size and is often used to search for the most likely cluster, and there are several options for reporting the secondary clusters. The default option for the Poisson model is based on the Gini coefficient among the hierarchical non-overlapping clusters with already reported clusters. More detailed procedures and other options are described in the SaTScan^™^ user guide 9.4 [7].

### Poisson model

The Poisson-based scan statistic, which is suitable for count data, can be used to identify the clusters of high (or low) incidence rates of events. Let *p* and *q* be the incidence rates of events within and outside scanning window *Z*. The likelihood ratio test statistic given *Z* for testing *H*_0_: *p* = *q* against *H*_*a*_: *p* > *q* is

$$\lambda_{Z}=\frac{{\left(\frac{c_{Z}}{n_{Z}}\right)}^{c_{Z}}{\left(\frac{C-c_{Z}}{N-n_{Z}}\right)}^{C-c_{Z}}}{{\left(\frac{C}{N}\right)}^{C}}I\left(\frac{c_{Z}}{n_{Z}}>\frac{C-c_{Z}}{N-n_{Z}}\right),$$

where *c*_*Z*_ and *n*_*Z*_ are the number of cases and population in scanning window *Z*, and C and N are the total number of cases and population in the study area. If we want to search for a cluster area that has a lower incidence rate of events, the indicator function $I\left(\frac{c_{Z}}{n_{Z}}>\frac{C-c_{Z}}{N-n_{Z}}\right)$ is replaced by $I\left(\frac{c_{Z}}{n_{Z}}<\frac{C-c_{Z}}{N-n_{Z}}\right)$.

Han *et al*. [13] showed that the size of a reported cluster is often close to the pre-defined maximum scanning window size using 2006 U.S. cancer mortality data when analyzed with an earlier version of SaTScan^™^. This suggests that a larger scanning window size has the potential to exaggerate the conclusions of the most likely cluster. In some cases, the cluster formed from the combination of small clusters in close proximity could have the largest likelihood ratio test statistic, despite including some non-informative areas with few events. Han *et al*. [13] proposed to use the Gini coefficient as a more intuitive and systematic way to determine the best collection of clusters to report.

The Gini coefficient is a measurement of income distribution equality developed by Gini [14]. It is usually defined as a summary measure on a Lorenz curve [19, 20], which shows the proportion of overall income (*y*%) assumed by the bottom *x*% of the population. The Gini coefficient can be calculated as two times the area between the reference line of *y* = *x* and the Lorenz curve. Han *et al*. [13] applied the methods of the Lorenz curve and the Gini coefficient to describe collections of disease clusters. The Lorenz curve for a cluster model was defined using the cumulative percentages of observed cases and expected cases on the *x*—and *y*-axes, respectively. If there is a significant cluster in the study region, the Lorenz curve would connect the three points of (0,0), (*x*_1_, *y*_1_), and (1,1), where *x*_1_ is the percentage of observed cases and *y*_1_ is the percentage of expected cases in the cluster. As *x*_1_ gets larger compared to *y*_1_, which means that more cases than the expected count are concentrated in the cluster, the Lorenz curve will get further away from the reference line of *y* = *x* and the Gini coefficient will be higher. The reference line indicates that the number of cases is proportional to the population (or the expected number of cases) for each region, and hence there are no significant clusters. When there are two or more significant clusters, they are sorted by their relative risks and the cumulative percentages of observed and expected cases are calculated in that order. The one with the highest Gini coefficient value among several competing collections of non-overlapping clusters is the best collection to report [13]. One should refer to the article by Han *et al*. [13] for more detailed information on the definition and calculation of the Gini coefficient in the Poisson-based spatial scan statistic.

In the SaTScan^™^ software, 15 different candidates for the maximum reported cluster sizes are considered to find the optimal value of the MRCS. For each candidate, the Gini coefficient based on detected significant clusters are calculated. Then, the one with the highest Gini coefficient value is assessed as the best collection to describe cluster patterns among the 15 candidates. Through the simulation studies and real cancer mortality data, Han *et al*. [13] showed that the Gini coefficient worked well for finding an appropriate MRCS. However, the performance of the method has been evaluated only for the Poisson model. To employ the Gini coefficient in other probability models, we need to develop a model-specific application.

### Gini coefficient for ordinal model

Now, we propose the application of the Gini coefficient for the ordinal model. First, we briefly review the spatial scan statistic for ordinal data proposed by Jung *et al*. [2] Suppose an ordinal outcome variable has *K* categories (*k* = 1, …, *K*) with the probability of category *k* inside and outside the scanning window *Z* being *p*_*k*_ and *q*_*k*_, respectively. Then, the null and alternative hypotheses are written as

$$H_{0}:\;p_{1}=q_{1},\ldots ,p_{K}=q_{K}\text{ for all }Z\text{ vs}.\;H_{a}:\;\frac{p_{1}}{q_{1}}\leq \frac{p_{2}}{q_{2}}\leq \ldots \leq \frac{p_{K}}{q_{K}}\;\text{for some }Z.$$

The order restriction in the alternative hypothesis, called the likelihood ratio ordering (LRO) [21], is used to find the clusters with high rates of the higher-valued category (e.g. more serious cancer stage). The likelihood ratio test statistic, given scanning window *Z* for the ordinal model, is defined as

$$\lambda_{Z}=\frac{\prod_{k}\left(\prod_{i\in Z}{\hat{p}}_{k}{}^{c_{ik}}\prod_{i\notin Z}{\hat{q}}_{k}{}^{c_{ik}}\right)}{\prod_{k}\prod_{i}{\hat{p}}_{0k}{}^{c_{ik}}}$$

where *c*_*ik*_ is the number of observations in region *i* and category *k*, ${\hat{p}}_{0k}=C_{k}/C\left(=\sum_{i}c_{ik}/\sum_{i,k}c_{ik}\right)$ is the maximum likelihood estimate (MLE) of *p*_*k*_ (= *q*_*k*_) under the null hypothesis, and ${\hat{p}}_{k}$ and ${\hat{q}}_{k}$ are MLEs of *p*_*k*_ and *q*_*k*_ under the alternative hypothesis. Note that some categories can be combined in the detected clusters. Details of the spatial scan statistic for ordinal data are explained in the paper by Jung *et al*. [2].

To define the Gini coefficient for the ordinal model, we need to consider how to represent the distribution of the categories for a cluster with high rates of the higher-valued category compared to the total observations. While constructing the Lorenz curve for the Poisson model is somewhat intuitive, it is rather not straightforward for the ordinal model. Suppose that there is only one significant cluster Z*. We define the *x*-coordinate of the point corresponding to the cluster on the Lorenz curve for the ordinal model as

$$\frac{\sum_{k}k\left({\hat{p}}_{k}\sum_{k}\sum_{i\epsilon Z^{*}}c_{ik}\right)}{\sum_{k}kC_{k}}$$ (1)

and the *y*-coordinate as

$$\frac{\sum_{k}\sum_{i\in Z^{*}}c_{ik}}{\sum_{k}C_{k}}.$$ (2)

That is, we define the Lorenz curve for the ordinal model so that the *x*-axis represents the cumulative proportions of weighted cases and the *y*-axis represents the cumulative proportions of total cases (observations). To reflect the order of the categories when calculating the weighted cases for the *x*-coordinate Eq (1), we assign ordinal scores to categories. Assigning scores to ordered categories is a common way to deal with ordinal data. Doing that treats the ordinal scale as an interval scale [22]. The denominator is simply the weighted number of total cases, which can be interpreted as the sum of attributes (represented by the ordered scores) that the total cases have. In the numerator, instead of weighting the observed number of cases by the score in the detected cluster, we use the estimated number of cases derived from the total number of observations multiplied by the ML estimates of *p*_*k*_ to avoid any confusion in case of the combined categories in detected clusters. The numerator can be interpreted as the sum of attributes that the cases in the cluster have. Therefore, the point (*x*, *y*) represents that *y*(×100)% of total observations have *x*(×100)% of total attributes. As the cluster has higher rates of the higher-valued category, the value for the *x*-coordinate gets larger and the Lorenz curve will get further away from the reference line, which will result in a higher value of the Gini coefficient, as in the Poisson model. When two or more significant clusters exist, we obtain each coordinate for each cluster by cumulating the numerators in Eqs (1) and (2) for clusters sorted by the statistical significance. If we have *J* points of (*x*_*j*_, *y*_*j*_)*j* = 1,…, *J* on the Lorenz curve for the multiple clusters, the Gini coefficient can be calculated as $\sum_{j=1}^{J+1}\left(x_{j-1}y_{j}-x_{j}y_{j-1}\right)$ with (*x*_0_, *y*_0_) = (0, 0) and (*x*_*J*+1_, *y*_*J*+1_) = (1, 1) as shown in the paper by Han *et al*. [13]. We select the one with the highest Gini coefficient value among several competing collections of non-overlapping clusters as the best collection to report.

**Fig 1:**
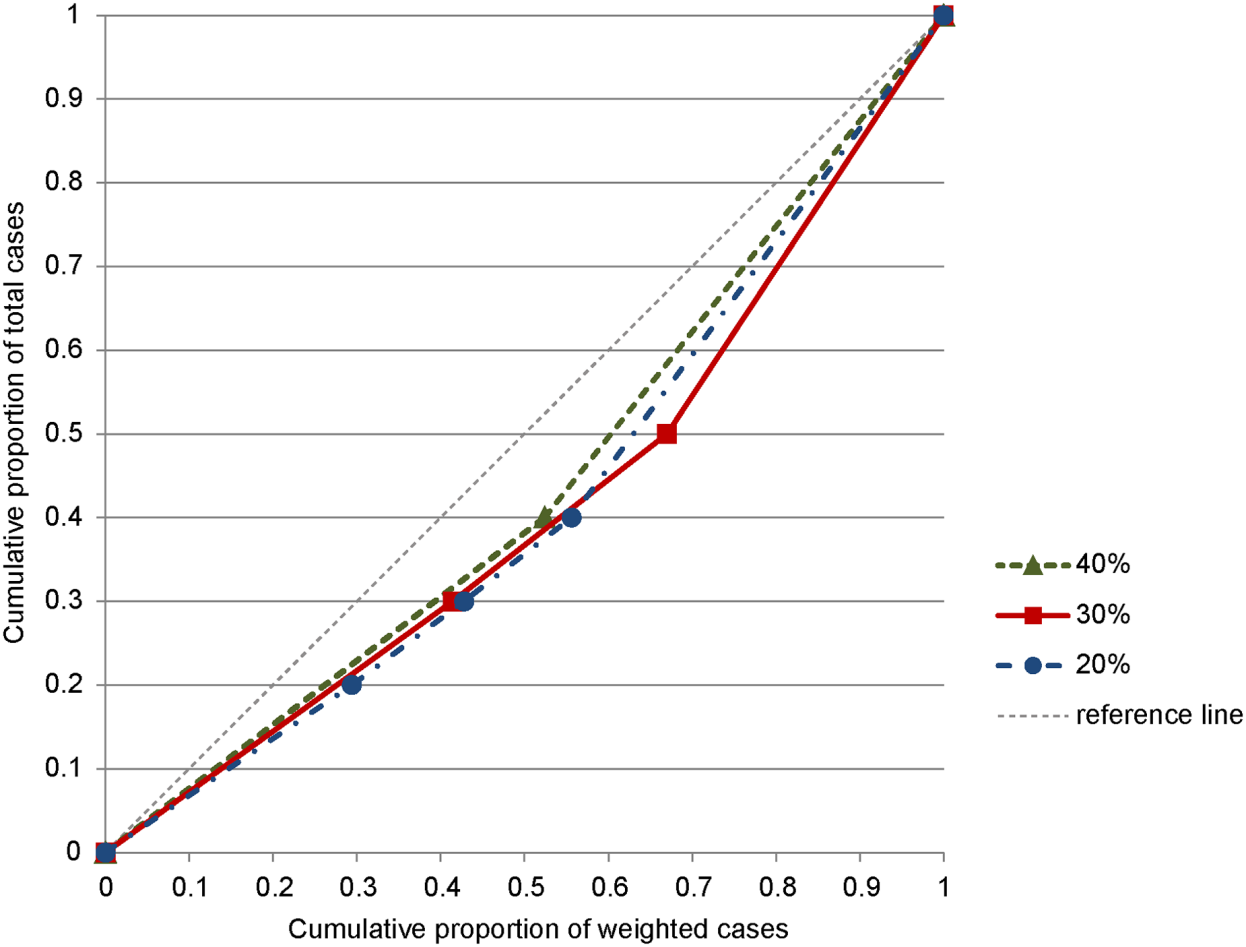
Lorenz curves for the ordinal model constructed from the hypothetical example of cluster detection analysis results for three different MRCS (see Table 1).

As an illustration to show how the Lorenz curve is constructed for the ordinal model, we presented a hypothetical example of cluster detection analysis results in Table 1 and the corresponding Lorenz curves in Fig 1. Suppose that a study area has 1000 cases in total with the same number of cases in four categories. If we obtain the cluster detection results for three different MRCS as listed in Table 1, we construct the corresponding three different Lorenz curves as shown in Fig 1. The Gini coefficient for each MRCS can be calculated two times the area between the reference line and each Lorenz curve. In this hypothetical example, we choose 30% as the optimal MRCS because the value of the Gini coefficient is the highest. Although the single most likely cluster reported at 40% of MRCS attains the highest value of the log-likelihood ratio (LLR) test statistic, it may not be the best cluster to report. We can see a lower proportion for category 1 and a higher proportion for category 4 in cluster 1 reported at 30% of MRCS compared with cluster 1 reported at 40% of MRCS. Cluster 1 at 30% of MRCS has higher rates of the higher-valued category, which pushes the Lorenz curve further away from the reference line and produces a higher value of Gini coefficient compared with cluster 1 at 40% of MRCS. The clusters reported at 30% of MRCS seem to be more meaningful and the best collection of clusters that shows distinct geographic pattern the best.

**Table 1:** A hypothetical example of cluster detection analysis results for three different MRCS and the values of the Gini coefficient (see Fig 1).

MRCS	Cluster	#Total cases	# Obs in each category	LLR	Gini
40%	1	400	(10, 50, 160, 180)	194.33	0.124
30%	1	300	(5, 35, 80, 180)	180.16	0.176
	2	200	(10, 30, 70, 90)	55.09	
20%	1	200	(5, 5, 40, 150)	173.21	0.163
	2	100	(5, 10, 30, 55)	35.13	
	3	100	(10, 10, 30, 50)	24.28	

^#^ Obs in each category, number of observations in each category; LLR, log-likelihood ratio.

### Simulation study

To evaluate the performances of the proposed method in the ordinal model, we conducted simulation studies with several cluster models using the geographic information of 25 districts of Seoul, Korea.

In the first cluster model, settings vary by the number of cases in the true cluster and alternative hypotheses. Assuming four categories for an ordinal outcome, we considered *H*_0_: ***p*** = ***q*** = (0.25, 0.25, 0.25, 0.25) as the null hypothesis and five different alternative hypotheses satisfying the LRO:

Scenario A: ***p*** = (0.10, 0.30, 0.30, 0.30)
Scenario B: ***p*** = (0.20, 0.20, 0.30, 0.30)
Scenario C: ***p*** = (0.20, 0.20, 0.20, 0.40)
Scenario D: ***p*** = (0.15, 0.25, 0.25, 0.35)
Scenario E: ***p*** = (0.15, 0.20, 0.25, 0.40)

The first four hypotheses were used in the simulation study in the paper by Jung *et al*. [2] and the last one was newly added here. The true clusters created under these scenarios have higher probabilities for higher categories compared to outside the clusters. For example, a cluster created under scenario A has a lower probability of category 1 (0.10 vs. 0.25) and a higher probability of categories 2 to 4 (0.30 vs. 0.25) compared to non-cluster areas.

**Fig 2:**
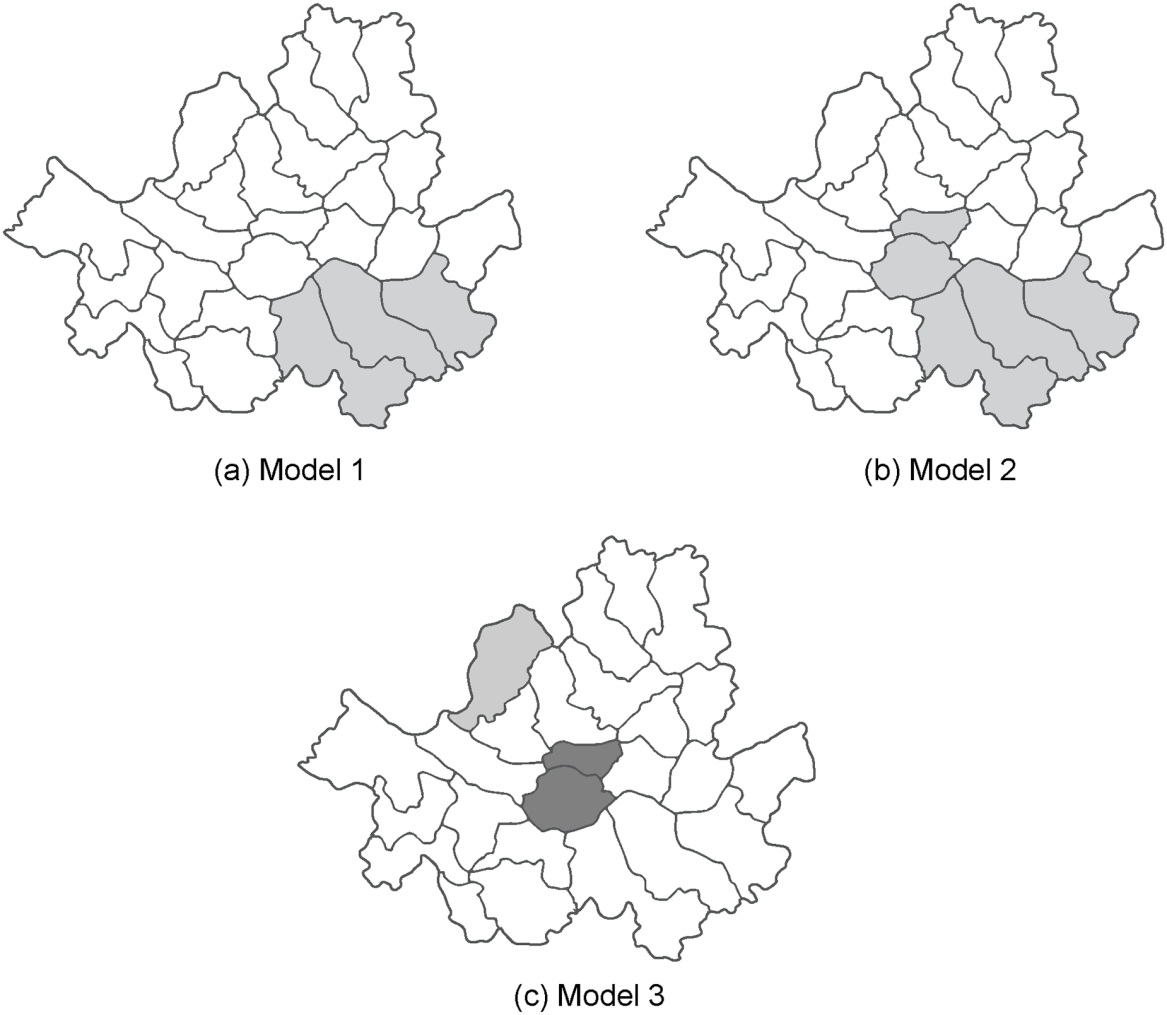
Three simulated cluster models. (a) model 1: a single circular cluster, (b) model 2: a single elliptic cluster, and (c) model 3: two clusters slightly apart from each other.

We set 2000 cases in the whole study region and a varying number of cases (200, 400 and 800, which are 10%, 20% and 40% of the total cases, respectively) in the true cluster of a circular shape, which comprises three districts (see Fig 2(a)). For the 15 combinations (five different hypotheses and three different numbers of cases), we generated 1000 random data sets and searched for clusters with high rates of high-valued categories using the circular scan statistic with 17 different MRCS (1, 2, 3, 4, 5, 6, 8, 10, 12, 15, 20, 25, 30, 35, 40, 45 and 50% of the total cases). Then, we calculated the values of the Gini coefficient for each candidate of maximum sizes for each data set when significant clusters were detected and summarized the frequency of the optimal maximum sizes chosen by the Gini coefficient (highest value) among 1,000 random data sets. For the chosen optimal MRCS, precision was also measured using the sensitivity and positive predicted value (PPV) based on detected clusters. The sensitivity is defined as the proportion of districts detected correctly among the districts in the true cluster, and PPV is the proportion of districts detected correctly among the districts in the detected cluster. Larger values of these measures indicate that the result is more precise in detecting the true cluster.

We also considered two different cluster models with various numbers of cases in the true cluster of different shapes. These models were evaluated with 10000 of the total number of cases only under scenario E of the null hypothesis *H*_0_: ***p*** = ***q*** = (0.25, 0.25, 0.25, 0.25) and the alternative *H*_*a*_: ***p*** = (0.15, 0.20, 0.25, 0.40). Fig 2(b) and 2(c) show the locations and the sizes of clusters for cluster models 2 and 3, respectively. We intended to create different types of cluster models from model 1 of a single cluster in a compact shape. The cluster in model 2 is of an irregular shape and two small clusters slightly apart from each other were created in model 3. In model 2, the cluster consists of 5 districts and three different numbers of cases (2000, 3000, and 4000) inside the cluster were considered. For cluster model 3, we set the locations of the two clusters close to each other but not connected with non-significant districts in between. There are 2000 cases in the deep gray areas (2 districts) and 1500 cases in the light gray areas (1 district). The method of evaluation was the same as in the first cluster model except that elliptical windows were additionally used (with default options for the shape, angle, and non-compactness parameter (medium penalty) in SaTScan^™^ [7]). For comparison, we included simulations for the default setting of SaTScan, which is 50% of MRCS, for all three cluster models.

## Results

### Simulation results

In the first cluster model, the Gini coefficient most often picked the optimal MRCS that was the same size as the true cluster except for one case (scenario B with 200 cases in the true cluster). In addition, the sensitivity and PPV were very high at the best-chosen maximum size. For example, the results for 800 cases (40% of total cases) show that 40% is the most chosen MRCS based on the Gini coefficient with very good accuracy (see Table 2). In addition, we found that the sensitivity decreases as the MRCS decreases below the best size. Conversely, PPV decreases as the MRCS increases above the best size. Such trends were somewhat expected because a smaller cluster would be reported with a lower maximum size and a large cluster would be reported with a higher size. Results for 400 cases (20% of total cases) were very similar to those for 800 cases (data are not shown). The sensitivity and PPV for the default setting were comparable to those for the most chosen MRCS. Still, higher accuracy was obtained at the best-chosen size.

**Table 2:** Simulation results of cluster model 1 (10% and 40% of the total cases in the true cluster). Maximum reported cluster sizes chosen by the Gini coefficient at least once are only shown. Cells most chosen as the optimal maximum size are shaded in gray.

	Maximum reported cluster size																
	2	3	4	5	6	8	10	12	15	20	25	30	35	40	45	50	Default
Scenario A																	
200 cases																	
# of OMRCS	0	3	0	36	40	109	729	5	36	24	15	2	1	0	0	0	
Sensitivity	-	0.67	-	0.65	0.64	0.77	1.00	0.80	0.99	0.99	1.00	1.00	1.00	-	-	-	0.96
PPV	-	1.00	-	1.00	0.98	0.98	1.00	0.65	0.75	0.58	0.50	0.43	0.38	-	-	-	0.97
800 cases																	
# of OMRCS	0	0	0	0	0	0	0	0	0	134	109	189	3	492	55	18	
Sensitivity	-	-	-	-	-	-	-	-	-	0.98	0.98	1.00	0.89	1.00	1.00	1.00	0.99
PPV	-	-	-	-	-	-	-	-	-	0.99	0.99	0.99	0.62	1.00	0.71	0.52	0.96
Scenario B																	
200 cases																	
# of OMRCS	26	120	17	16	15	31	2	19	20	23	16	9	11	8	11	12	
Sensitivity	0.17	0.33	0.10	0.23	0.33	0.65	0.00	0.61	0.70	0.74	0.92	1.00	0.78	0.92	0.88	0.89	0.72
PPV	0.50	0.94	0.29	0.66	1.00	0.95	0.00	0.61	0.57	0.49	0.46	0.42	0.28	0.28	0.25	0.22	0.56
800 cases																	
# of OMRCS	0	0	0	0	0	0	1	5	0	0	4	41	85	442	191	231	
Sensitivity	-	-	-	-	-	-	0.33	0.33	-	-	0.33	0.67	0.67	0.99	1.00	1.00	0.94
PPV	-	-	-	-	-	-	1.00	1.00	-	-	1.00	1.00	0.86	0.99	0.68	0.51	0.82
Scenario C																	
200 cases	0	9	3	11	14	180	598	13	60	60	28	11	5	3	2	3	
# of OMRCS	-	0.41	0.33	0.52	0.36	0.67	1.00	0.69	0.94	0.97	1.00	1.00	1.00	1.00	0.83	1.00	0.90
Sensitivity	-	1.00	0.67	1.00	0.96	0.99	1.00	0.63	0.73	0.59	0.49	0.42	0.35	0.30	0.22	0.24	0.92
PPV																	
800 cases																	
# of OMRCS	0	0	0	0	0	0	0	0	0	12	10	94	11	717	111	45	
Sensitivity	-	-	-	-	-	-	-	-	-	0.67	0.70	0.97	0.79	1.00	1.00	1.00	0.99
PPV	-	-	-	-	-	-	-	-	-	1.00	0.87	0.98	0.89	1.00	0.71	0.52	0.94
Scenario D																	
200 cases																	
# of OMRCS	0	23	3	14	15	214	450	26	78	71	45	24	13	8	8	8	
Sensitivity	-	0.33	0.33	0.43	0.38	0.67	0.99	0.67	0.93	0.95	0.99	0.99	1.00	1.00	0.96	0.96	0.81
PPV	-	1.00	0.67	1.00	0.97	0.99	1.00	0.66	0.71	0.59	0.48	0.42	0.35	0.31	0.27	0.24	0.83
800 cases																	
# of OMRCS	0	0	0	0	0	0	0	0	0	7	8	55	16	658	153	103	
Sensitivity	-	-	-	-	-	-	-	-	-	0.67	0.67	0.92	0.71	1.00	1.00	1.00	0.99
PPV	-	-	-	-	-	-	-	-	-	1.00	0.92	0.98	0.96	1.00	0.70	0.53	0.92
Scenario E																	
200 cases																	
# of OMRCS	0	1	0	29	25	130	684	9	47	40	18	5	5	6	1	0	
Sensitivity	-	0.33	-	0.59	0.57	0.71	1.00	0.67	0.95	1.00	1.00	1.00	1.00	1.00	1.00	-	0.92
PPV	-	1.00	-	0.95	1.00	1.00	1.00	0.67	0.74	0.60	0.49	0.43	0.37	.31	0.27	-	0.94
800 cases																	
# of OMRCS	0	0	0	0	0	0	0	0	0	36	19	37	4	767	115	22	
Sensitivity	-	-	-	-	-	-	-	-	-	1.00	0.97	0.97	0.83	1.00	1.00	1.00	1.00
PPV	-	-	-	-	-	-	-	-	-	1.00	0.97	1.00	0.75	1.00	0.71	0.52	0.95

^#^ of OMRCS, frequency chosen as the optimal maximum reported cluster size by the Gini coefficient among 1000 random data sets; PPV, positive predictive value.

When the true clusters were irregularly shaped (cluster model 2), the best-chosen maximum size was smaller than the true cluster size using either circular or elliptic windows. The Gini coefficient most often picked 6, 8, and 12% as the optimal sizes when 20, 30, and 40% of the total cases were in the true cluster, respectively (see Table 3). Although the Gini coefficient picked a smaller MRCS than the true cluster size, the clusters were detected with almost perfect accuracy. When using the Gini coefficient, multiple significant clusters, which are contiguously located and compose the true cluster, were found. We considered the true cluster to be found if the five districts in the cluster were exactly identified as a single cluster or as separate clusters, because both results are indistinguishable. Using elliptic windows, the Gini coefficient chose the same size as the true cluster size as the optimal MRCS 40, 46, and 69 times out of 1000 replications with high accuracy when 20, 30, and 40% of the total cases were in the true cluster, respectively. Using circular windows, however, the true cluster could never be accurately found as a single cluster because of the cluster shape. The Gini coefficient almost always chose smaller values of MRCS as the optimal size than the true cluster size. When using circular windows, the PPV was always lower at the default setting than at the optimal MRCS, which implies that the default setting reports clusters larger than the true ones.

**Table 3:** Simulation results of cluster model 2 (20%, 30%, and 40% of the total cases in the true cluster of irregular shape). Maximum reported cluster sizes chosen by the Gini coefficient at least once are only shown. Cells most chosen as the optimal maximum size are shaded in gray.

	Maximum reported cluster size													
	5	6	8	10	12	15	20	25	30	35	40	45	50	Default
Circular shape														
2000 cases														
# of OMRCS	28	554	291	10	9	98	0	0	10	0	0	0	0	
Sensitivity	1.00	1.00	1.00	0.98	1.00	1.00	-	-	1.00	-	-	-	-	0.99
PPV	1.00	1.00	1.00	0.77	0.78	1.00	-	-	0.71	-	-	-	-	0.74
3000 cases														
# of OMRCS			556	27	317	8	92	0	0	0	0	0	0	
Sensitivity			1.00	1.00	1.00	1.00	1.00	-	-	-	-	-	-	0.99
PPV			1.00	1.00	1.00	0.5	1.00	-	-	-	-	-	-	0.72
4000 cases														
# of OMRCS	0	0	0	29	536	231	101	57	36	0	0	0	0	
Sensitivity	-	-	-	1.00	1.00	1.00	1.00	1.00	1.00	-	-	-	-	1.00
PPV	-	-	-	1.00	1.00	1.00	0.99	0.99	1.00	-	-	-	-	0.71
Elliptic shape														
2000 cases														
# of OMRCS	37	672	104	35	5	102	40	5	0	0	0	0	0	
Sensitivity	0.99	0.99	1.00	0.98	0.92	0.99	1.00	1.00	-	-	-	-	-	0.97
PPV	1.00	1.00	0.92	0.85	1.00	0.87	1.00	0.83	-	-	-	-	-	0.99
3000 cases														
# of OMRCS	0	0	663	32	105	14	123	11	46	6	0	0	0	
Sensitivity	-	-	1.00	1.00	1.00	1.00	1.00	1.00	0.99	1.00	-	-	-	0.99
PPV	-	-	1.00	1.00	0.86	0.78	0.96	0.78	1.00	0.83	-	-	-	0.99
4000 cases														
# of OMRCS	0	0	0	33	557	217	16	73	32	0	69	3	0	
Sensitivity	-	-	-	1.00	1.00	1.00	0.99	1.00	1.00	-	1.00	1.00	-	1.00
PPV	-	-	-	1.00	1.00	0.98	0.85	0.96	0.81	-	1.00	0.83	-	0.97

^#^ of OMRCS, frequency chosen as the optimal maximum reported cluster size by the Gini coefficient among 1000 random data sets; PPV, positive predictive value.

For cluster model 3, the Gini coefficient generally picked the optimal MRCS the same as the cluster size of either one of the two (15% or 20% of total observations) as shown in Table 4. In such cases, the districts in the true clusters were correctly identified. Both the sensitivity and PPV were equal to 1. We observed a very low PPV at the default setting when using either circular or elliptic windows. The default setting often reported a single large cluster including two true clusters with non-cluster areas in between.

**Table 4:** Simulation results of cluster model 3 (15% and 20% of the total cases in each of two clusters slightly apart from each other). Maximum reported cluster sizes chosen by the Gini coefficient at least once are only shown. Cells most chosen as the optimal maximum size are shaded in gray.

	Maximum reported cluster size								
	15	20	25	30	35	40	45	50	Default
Circular shape									
# of OMRCS	942	57	0	0	0	1	0	0	
Sensitivity	1.00	1.00	-	-	-	1.00	-	-	1.00
PPV	1.00	1.00	-	-	-	0.38	-	-	0.49
Elliptic shape									
# of OMRCS	940	49	0	0	0	11	0	0	
Sensitivity	1.00	1.00	-	-	-	1.00	-	-	1.00
PPV	1.00	1.00	-	-	-	0.68	-	-	0.66

^#^ of OMRCS, frequency chosen as the optimal maximum reported cluster size by the Gini coefficient among 1000 random data sets; PPV, positive predictive value.

### Application to real data

To demonstrate the utility of the Gini coefficient in the ordinal model, we used the birth order data in Seoul, Korea for 2013. The birth order was categorized as the first, second, or third child and higher. The data set was based on birth certificate registration provided by the Korean Statistical Information Service (KOSIS). Ethics approval was not required because we used anonymized data publicly available from the KOSIS website (kosis.kr). We aggregated the data into 25 districts in Seoul. There were 83848 registry cases in total with 48248 (57.5%), 29656 (35.4%), and 5944 (7.1%) cases for each birth order category. To search for clusters with high rates of higher birth order, we used the spatial scan statistic for ordinal data and determined the optimal MRCS based on the Gini coefficient. Both circle and ellipse were used as the scanning window shape.

**Fig 3:**
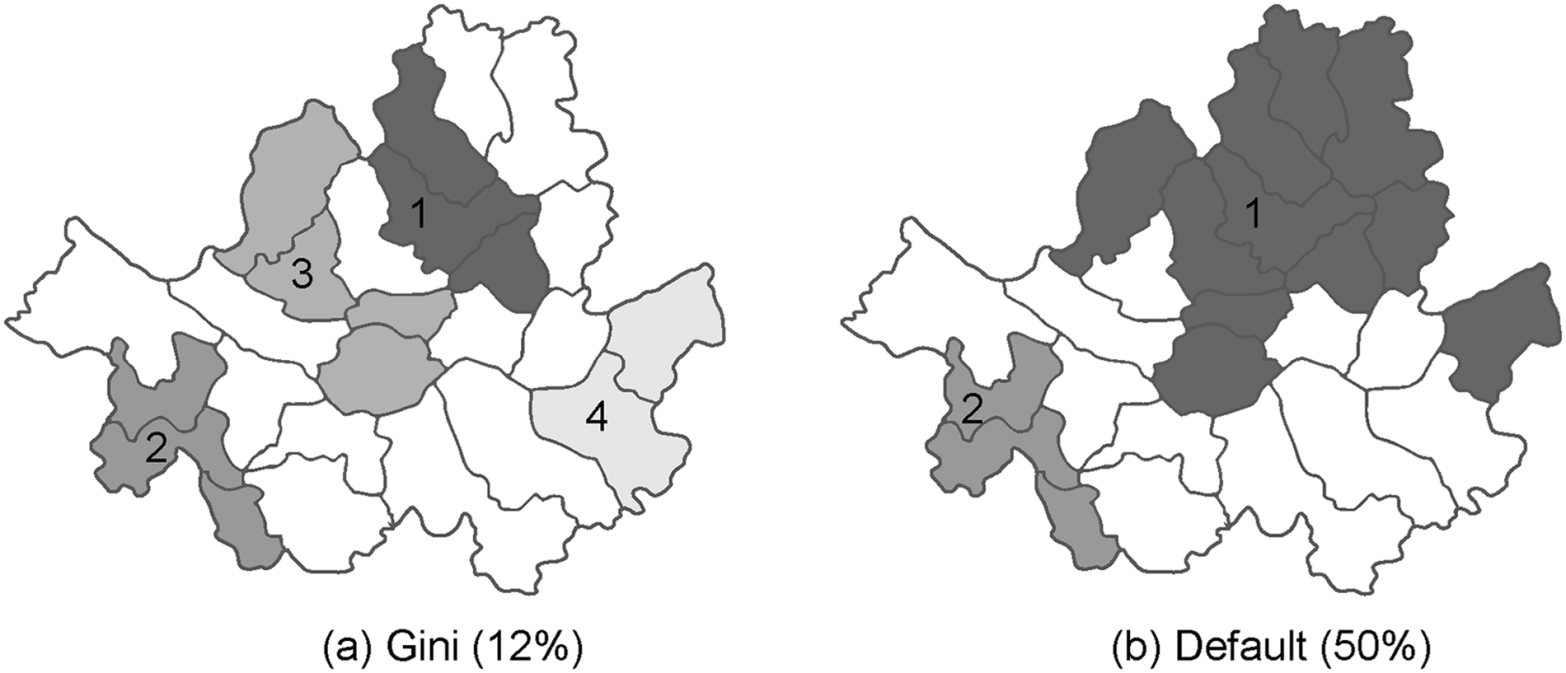
Clusters with high rates of higher birth order category in Seoul, Korea identified using (a) the Gini coefficient (12% of MRCS) and (b) the default setting (50% of MRCS).

When using the circular scanning windows, the Gini coefficient picked 30% as the optimal MRCS, and three significant clusters were identified. This was consistent with the result based on the default size of 50%. In this case, the most likely cluster contained 29.1% of the total cases. However, the results using the elliptic windows were quite different. Two clusters were detected using the default size (50%). The most likely cluster included 34% of the total cases. However, the Gini coefficient picked 12% as the optimal MRCS, and four clusters were detected. Some districts of three of these clusters (cluster 1, 3, and 4 in Fig 3(a)) overlapped with the most likely cluster identified using the default size (cluster 1 in Fig 3(b)). Table 5 lists detailed information on the detected clusters. Even though we do not know which cluster pattern is true, the trend of high rates of higher birth order in the most likely cluster based on the Gini coefficient (0.546, 0.374, 0.080) seems somewhat clearer than in the most likely cluster identified using the default (0.555, 0.368, 0.077), when compared to the observed proportions of the three categories in the whole study area (0.575, 0.354, 0.071). The observed proportions of the three categories in the 4 districts excluded from cluster 1 of the default setting when using the Gini coefficient were (0.565, 0.364, 0.071), which are very similar to those in the whole study area. Cluster 1 reported at the default setting seem to include areas with non-elevated rates.

**Table 5:** Cluster detection analysis results for birth order data in Seoul, Korea using the elliptic window shape (see Fig 2).

MRCS	Cluster	# Districts	# Obs in each category	LLR	*p*-value
12% (chosen by Gini)	1	3	(4894, 3348, 720)	19.23	0.001
	2	3	(5352, 3642, 737)	14.54	0.001
	3	4	(5554, 3287, 794)	10.52	0.013
	4	2	(5565, 3719, 729)	8.95	0.024
50%	1	9	(15806, 10493, 2192)	40.00	0.001
	2	3	(5352, 3642, 737)	14.54	0.001

^#^ Districts, number of districts;

^#^ Obs in each category, number of observations in each category; LLR, log-likelihood ratio.

## Discussion

Our simulation study results imply that we might find a larger cluster than the true cluster when using the default MRCS (50% of the total cases). The PPV was always lower at the default setting than at the best-chosen MRCS by the Gini coefficient. In addition, if we choose the MRCS arbitrarily, detected clusters could be different from the true cluster patterns. For a compact single cluster, the Gini coefficient most often picked the same size of the true cluster as the optimal MRCS with high accuracy. However, when the true clusters were irregularly shaped or located slightly apart from each other, the Gini coefficient tended to choose a smaller MRCS than the true clusters, which gives several smaller clusters. Still, the detected clusters based on the Gini coefficient had a high sensitivity and PPV. Using the Gini coefficient developed specifically for the ordinal model can identify a more refined collection of non-overlapping clusters to report for ordinal data.

The simulation results of cluster model 1 from the five different scenarios seem very similar except for scenario B. The distribution of the chosen optimal MRCS was spread wider over almost all candidates of MRCS for 200 cases than that for 800 cases. We think that such phenomena were related to the strength of the true cluster and statistical power for each alternative hypothesis. As seen in the paper by Jung *et al*. [2], power for scenario B was relatively low compared to the other scenarios. That is why the frequencies chosen as the optimal maximum at the true cluster size were lower for scenario B. Also, it is natural that the cluster is more clearly differentiated from non-cluster areas when there are more cases in the cluster. The Gini coefficient chose the true cluster size as the best MRCS more often for 800 cases in the cluster than for 200 cases.

Compared to the simulation results of cluster model 1, sensitivity and PPV at the chosen values of MRCS were very high in the results for cluster models 2 and 3. That might be due to higher numbers of cases in the cluster and total observations in the whole study area compared to those for cluster model 1. An important point here is that the Gini coefficient almost always picked a smaller size as the best MRCS than the true cluster size when the cluster is not in a circular shape and made it possible to report clusters very precisely. Using circular windows, an irregularly shaped true cluster could never be found as a single cluster with high accuracy. A single circular cluster would include either non-significant neighbors with the true cluster areas or only a part of the true cluster.

We think that using the Gini coefficient for optimizing the MRCS might also work well for finding irregularly shaped clusters. The Gini coefficient seems to produce multiple small clusters, detected separately when the true cluster is not in a circular shape, but the identified clusters are contiguously located, which in turn, can be regarded as a single cluster. Using an irregularly shaped scanning window in various ways has been proposed in several studies [23–26]. Recently, Kim and Jung [27] evaluated the Gini coefficient for detecting irregularly shaped clusters for the Poisson model. They conducted a simulation study assuming various types of cluster models in irregular shape. Their simulation study results showed that using the Gini coefficient worked better than the original spatial scan statistic for identifying irregularly shaped clusters, by reporting an optimized and refined collection of clusters rather than a single larger cluster. Further simulation studies should be conducted to evaluate the usefulness of finding irregularly shaped clusters using the Gini coefficient for the ordinal model as compared to using irregularly shaped scanning windows.

One may worry about the computational burden for calculation of the Gini coefficient at different MRCS. As we explained in Introduction section, we do not rerun the analysis multiple times using different values for the MSWS. We first evaluate all possible candidate windows at a larger value of MSWS (e.g., 50%) and then determine which clusters to report. We only need to filter clusters at different MRCS and perform simple algebraic calculations of the Gini coefficient. The additional computational burden to estimate the best MRCS using the Gini coefficient is minimal.

Optimizing the MRCS is an important issue in spatial scan statistics for properly and informatively discovering cluster patterns. Application of the Gini coefficient has been evaluated only for the Poisson and ordinal models. It would be an interesting research topic to employ such a criterion or newly developed measures to other models such as the multinomial, normal, and exponential models.

## Conclusions

In this paper, we presented the application of the Gini coefficient proposed by Han *et al*. [13] for the Poisson model to the spatial scan statistic for ordinal data to optimize the MRCS. With some sophisticated modification, the Gini coefficient can be effectively employed for the ordinal model. Through a simulation study and a real data example, we showed that the Gini coefficient can be successfully used to optimize the MRCS in the spatial scan statistic for ordinal data. The Gini coefficient for the Poisson model has already been implemented in SaTScan^™^. It can be consistently implemented for the ordinal model as well.

## Supporting information

S1 File Centroid information and case data of birth order in Seoul, Korea for 2013. (XLSX)

## References

[1] Kulldorff M. A spatial scan statistic. Communications in Statistics-Theory and methods 1997; 26(6):1481–1496.

[2] Jung I, Kulldorff M, Klassen AC. A spatial scan statistic for ordinal data. Statistics in Medicine 2007; 26(7):1594–1607. doi: 10.1002/sim.2607 16795130 10.1002/sim.2607

[3] Cook AJ, Gold DR, Li Y. Spatial cluster detection for censored outcome data. Biometrics 2007; 63(2):540–549. doi: 10.1111/j.1541-0420.2006.00714.x 17688506 10.1111/j.1541-0420.2006.00714.x

[4] Jung I, Kulldorff M, Richard OJ. A spatial scan statistic for multinomial data. Statistics in Medicine 2010; 29(18):1910 doi: 10.1002/sim.3951 20680984 10.1002/sim.3951 PMC4147837

[5] Kulldorff M, Huang L, Konty K. A scan statistic for continuous data based on the normal probability model. International journal of health geographics 2009; 8:58 doi: 10.1186/1476-072X-8-58 19843331 10.1186/1476-072X-8-58 PMC2772848

[6] Huang L, Tiwari RC, Zou Z, Kulldorff M, Feuer EJ. Weighted normal spatial scan statistic for heterogeneous population data. Journal of the American Statistical Association 2009; 104(487):886–898.

[7] Kulldorff M. SaTScan™ User Guide. In SaTScan™ User Guide.

[8] Goujon-Bellec S, Demoury C, Guyot-Goubin A, Hémon D, Clavel J. Detection of clusters of a rare disease over a large territory: performance of cluster detection methods. International journal of health geographics 2011; 10:53 doi: 10.1186/1476-072X-10-53 21970516 10.1186/1476-072X-10-53 PMC3204219

[9] Grubesic TH, Wei R, Murray AT. Spatial Clustering Overview and Comparison: Accuracy, Sensitivity, and Computational Expense. Annals of the Association of American Geographers 2014; 104(6):1134–1156.

[10] Huang L, Pickle LW, Das B. Evaluating spatial methods for investigating global clustering and cluster detection of cancer cases. Statistics in Medicine 2008; 27(25):5111–5142. doi: 10.1002/sim.3342 18712778 10.1002/sim.3342 PMC2575694

[11] Tango T, Takahashi K. A flexibly shaped spatial scan statistic for detecting clusters. International journal of health geographics 2005; 4:11 doi: 10.1186/1476-072X-4-11 15904524 10.1186/1476-072X-4-11 PMC1173134

[12] Ribeiro SHR, Costa MA. Optimal selection of the spatial scan parameters for cluster detection: a simulation study. Spatial and spatio-temporal epidemiology 2012; 3(2):107–120. doi: 10.1016/j.sste.2012.04.004 22682437 10.1016/j.sste.2012.04.004

[13] Han J, Zhu L, Kulldorff M, Hostovich S, Stinchcomb DG, Tatalovich Z, et al Using Gini coefficient to determining optimal cluster reporting sizes for spatial scan statistics. International journal of health geographics 2016; 15:27 doi: 10.1186/s12942-016-0056-6 27488416 10.1186/s12942-016-0056-6 PMC4971627

[14] Gini C. Variabilità e mutabilità. Reprinted in Memorie di metodologica statistica (Ed. Pizetti E, Salvemini, T). Rome: Libreria Eredi Virgilio Veschi 1912.

[15] Bell N, Kruse S, Simons RK, Brussoni M. A spatial analysis of functional outcomes and quality of life outcomes after pediatric injury. Injury Epidemiology 2014; 1:16 doi: 10.1186/s40621-014-0016-1 26613070 10.1186/s40621-014-0016-1 PMC4648946

[16] Fuchs S, Ornetsmu¨ ller C, Totschnig R. Spatial scan statistics in vulnerability assessment: an application to mountain hazards. Natural Hazards 2012; 64:2129–2151.

[17] Westercamp N, Moses S, Agot K, Ndinya-Achola JO, Parker C, Amolloh KO, et al Spatial distribution and cluster analysis of sexual risk behaviors reported by young men in Kisumu, Kenya. International Journal of Health Geographics 2010; 9:24 doi: 10.1186/1476-072X-9-24 20492703 10.1186/1476-072X-9-24 PMC2881901

[18] Dwass M. Modified randomization tests for nonparametric hypotheses. The Annals of Mathematical Statistics 1957; 28(1):181–187.

[19] Lorenz MO. Methods of measuring the concentration of wealth. Publications of the Americal Statistical Association 1905;9(70):209–219.

[20] Gastwirth JL. The estimation of the Lorenz curve and Gini index. The Review of Economics and Statistics 1972;54(3):306–316.

[21] Dykstra R, Kochar S, Robertson T. Inference for likelihood ratio ordering in the two-sample problem. Journal of the American Statistical Association 1995; 90(431):1034–1040.

[22] Agresti A. Analysis of Ordinal Categorical Data (2^nd^ edition). John Wiley & Sons, Inc 2010.

[23] Duczmal L, Assunção R. A simulated annealing strategy for the detection of arbitrarily shaped clusters. Computational Statistics & data Analysis 2004; 45(2):269–286.

[24] Patil GP, Taillie C. Upper level set scan statistic for detecting arbitrarily shaped hotspots. Environmental and Ecological Statistics 2004; 11(2):183–197.

[25] Duczmal L, Cançado ALF, Takahashi RHC, Bessegato LF. A genetic algorithm for irregularly shaped spatial scan statistics. Computational Statistics & data Analysis 2007; 52:(1):43–52.

[26] Tango T, Takahashi K. A flexible spatial scan statistic with a restricted likelihood ratio for detecting disease clusters. Statistics in Medicine 2012; 31(30): 4207–4218. doi: 10.1002/sim.5478 22807146 10.1002/sim.5478

[27] Kim J, Jung I. Evaluation of the Gini coefficient in spatial scan statistics for detecting irregularly shaped clusters. PLoS ONE 2017; 12(1):e0170736 doi: 10.1371/journal.pone.0170736 28129368 10.1371/journal.pone.0170736 PMC5271318

